# Retraction: A 1 year course of starch- and sucrose-reduced diet used by irritable bowel syndrome patients with diarrhoea and the effect of genetic variants

**DOI:** 10.3389/fnut.2024.1390602

**Published:** 2024-03-04

**Authors:** Frontiers Editorial Office

Following publication, the authors contacted the Editorial Office to request the retraction of the cited article, stating that they became aware of a technical issue in the sequencing protocol and the pipeline used for detecting DNA variants which affects the analysis of candidate genes rendering it inaccurate. For this reason, the study section focused on SI and other genes is to be considered unreliable, and its interpretation and drawn conclusions are unsupported. An investigation was conducted in accordance with Frontiers' policies that confirmed the findings reported in the article are no longer supported by the data and analyses; therefore, the article is retracted.

The authors concur with the retraction and sincerely regret any inconvenience this may have caused to the reviewers, editors, and readers of Frontiers in Nutrition.

